# Differences in trauma team activation criteria among Norwegian hospitals

**DOI:** 10.1186/1757-7241-18-21

**Published:** 2010-04-20

**Authors:** Kristin T Larsen, Oddvar Uleberg, Eirik Skogvoll

**Affiliations:** 1 Faculty of Medicine, Norwegian University of Science and Technology, Trondheim, Norway; 2 Department of Anaesthesia and Emergency Medicine, St Olav's University Hospital, Trondheim, Norway; 3 Institute for Circulation and Medical Imaging, Faculty of Medicine, Norwegian University of Science and Technology, Trondheim, Norway

## Abstract

**Background:**

To ensure the rapid and correct triage of patients in potential need of specialized treatment, Norwegian hospitals are expected to establish trauma teams with predefined criteria for their activation. The objective of this study was to map and describe the criteria currently in use.

**Methods:**

We undertook a cross-sectional survey in the summer of 2008, using structured telephone interviews to all Norwegian hospitals that might admit severely injured patients.

**Results:**

Forty-nine hospitals were included, of which 48 (98%) had a trauma team and 20 had a hospital-based trauma registry. Criteria for trauma team activation were found at 46 (94%) hospitals. No single criterion was common to all hospitals. The median number of criteria per hospital was 23 (range 8-40), with a total number of 156 and wide variation with respect to physiological "cut-off" values. The mechanism of injury was commonly in use despite a well-known, large over-triage rate.

**Conclusions:**

In recent years, Norwegian hospitals have gradually established trauma teams and criteria for their activation. These criteria show considerable variation, including physiological "cut-off" values.

## Background

Traumatic injury is well recognized as one of the main challenges in modern health care [1]. Worldwide, approximately 11.9 million people die annually as a result of trauma and thousands more are temporarily and permanently disabled [1]. Based on the lessons of war, civilian trauma systems have developed substantially within the last 50 years [2].

The first civilian trauma centers in the US (established in San Francisco and Chicago in 1966) and the landmark paper "Optimal hospital resources for care of the seriously injured," published in 1976 by the American College of Surgeons Committee on trauma (ACS-COT), marked a new era of structured trauma care [3,4]. Subsequent revisions of the paper by ACS-COT have followed as new knowledge evolves through systematic research, practical lessons learned, and technological developments [5-10].

Every injured patient should be treated as soon as possible at the right level of care. Organized trauma care systems, encompassing medical treatment from pre-hospital involvement to completed rehabilitation, significantly reduce injury-related mortality and morbidity in patients with moderate to severe injury [11-13].

The ideal system has been debated but should ensure appropriate patient care from resuscitation to rehabilitation. This includes triage guidelines in the field, adequate emergency medical services, and regional classification of hospitals according to the level of care [4,14].

At the scene of an accident, it may be difficult to identify patients with potentially serious injuries due to the diversity of patients, injuries, and the degree of physiological derangement. In 1986, the American College of Surgeons published a "Field Triage Decision Scheme" which was intended to guide pre-hospital care providers to transport injured patients to the most appropriate medical facility [2,5]. Initial experience led to recognition of inadequate triage, resulting in under- and over-triage at many trauma center facilities [2].

Many of these criteria have since been partially adopted for in-hospital use to perform trauma team activation (TTA). TTA shortens the time from when the patient arrives at the hospital until he or she is prepared in the operating room and improves the survival of severely injured patients [13]. Ideal criteria should be both 100% sensitive (identifying all seriously injured patients, i.e., yielding no under-triage) and 100% specific (yielding no over-triage). Over-triage rates up to 50% have been accepted to minimize unfavorable under-triage [9]. However, over-triage may result in an inadequate use of resources, increased workload, and longer out-of-hospital times [15]. In 2006, an expert panel published the report "Trauma system in Norway - Suggestions for organizing the treatment of severely injured patients" [16]. They concluded that a lack of systematic registration and national guidelines potentially cause suboptimal trauma care [16].

The aims of the present study were to investigate and compare the current use of TTA criteria in Norwegian hospitals.

## Methods

### Study

We conducted a cross-sectional survey with structured telephone interviews to all Norwegian hospitals receiving potentially severely injured patients. Interviews were performed from April to August 2008. Eligible contact persons responded to a structured questionnaire (Table 1) and provided a list describing the hospital TTA, if any. Contact persons were considered eligible if listed as 1) the hospital's BEST (Better and systematic trauma care - Foundation) contact person [17,18], 2) the Emergency Department head nurse, or 3) the head of the hospital Trauma Committee. Results from key questions in this survey are presented and discussed.

**Table 1:** Semi-structured questionnaire

Questions	Answer alternatives
**Type of hospital**	Local, Central, or Regional

**Does the hospital have acute surgical function?**	Yes or No

Has the hospital performed trauma team training according to the BEST^1 ^guidelines?	Yes or No

If yes, when was the first training?	Date

Have you been training during the last 12 months?	Yes or No

**Does the hospital have a trauma manual or other written guidelines for trauma treatment?**	Yes or No

**Does the hospital have a defined trauma team?**	Yes or No

**If yes, does the hospital have predefined criteria concerning when to perform TTA?**	If yes, which criteria does the hospital use today?

**How were these criteria developed?**	

Have the criteria been revised?	If yes, when was the last revision?

**If you do not have written criteria for TTA, how do you decide whether to activate the trauma team?**	

**Does the hospital have a trauma registry and/or an injury registry?**	Yes or No If yes: trauma registry and/or injury registry

Do you perform regular trauma meetings discussing trauma patients treated by the hospital?	If yes, how often?

Does the hospital have predefined criteria for transfer of trauma patients to higher level treatment facilities?	If yes, which criteria?

Do you plan to change your practice?	If yes, how and when?

**Marked questions are presented as results**	

^1^BEST: Better and systematic trauma care -- Foundation

The criteria were classified by subject matter or substantial interpretation by the author collecting and processing the data. If two criteria had different wording but only one interpretation, they were combined into one criterion. For instance "Penetrating injury" was specified by different hospitals as truncal, central, proximal to ankles and wrists, indicated by specific body parts, or unspecified. To allow a comparison of the criteria sets, these criteria were either classified as "centrally penetrating injury" or simply "penetrating injury". Criteria were assumed to relate to adults unless otherwise specified.

The regional ethics committee was informed about the study and decided that formal ethical approval was not required.

### Clinical setting

Norway is a narrow but long country covering 324,000 km^2^, with a straight-line distance from north to south of 1,800 km and a population of 4.8 million [19]. The scattered population (16 per km^2^) leads to challenges regarding patient transport and availability of specialized treatment [16,19].

The emergency medical service is well developed, with a combined ground and air ambulance service. Ambulance paramedics and general practitioners provide basic pre-hospital care, and the air ambulance service (with an anesthesiologist/paramedic crew) delivers advanced pre-hospital care. The latter responds separately when needed [16,20]. Hospitals are organized in a three-level system of local, central, and regional university hospitals [16]. Populations covered by local and central hospitals range from 13,000 to 400,000. University hospitals serve as trauma referral centers and cover populations varying in size from 250,000 to 2,500,000 [16].

## Results

**Figure 1:**
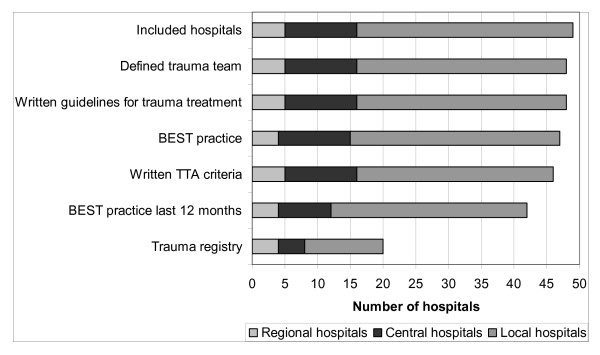
**Distribution of general results**.

**Figure 2:**
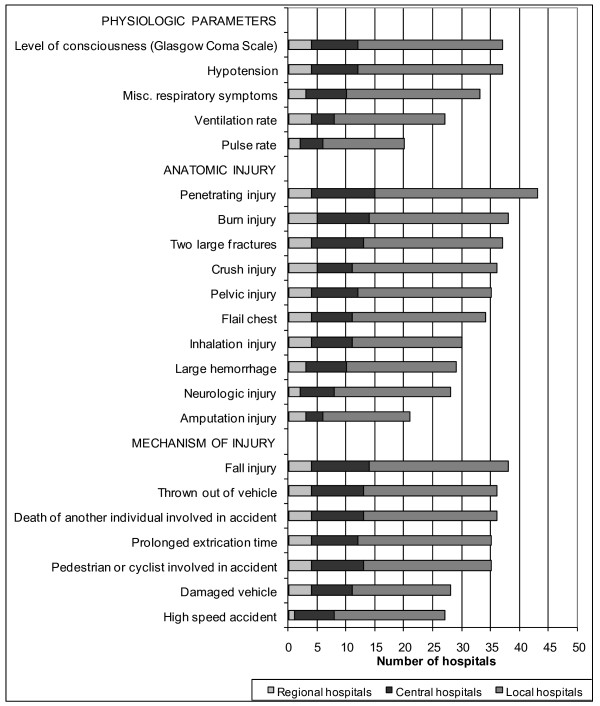
**Distribution of criteria most frequently in use, according to physiology, anatomy, and mechanism of injury**.

Forty-nine hospitals were included in this study. Five regional university hospitals, 11 central hospitals, and 33 local hospitals confirmed receiving potentially severely traumatized patients. Among these, 48 hospitals (98%) had a defined trauma team. Most of these (N = 46, 96% of hospitals with a trauma team) had predefined, written TTA criteria. One hospital had no trauma team due to a staff shortage. Two local hospitals had a trauma team but no specific criteria for activation. In one of these two hospitals, the surgeon on call or coordinating nurse in the emergency department assumed responsibility for activating the trauma team. A trauma registry was reported to be in operation at 20 hospitals. An overview of the general results is shown in Figure 1. The median number of criteria per hospital was 23 (range 8 - 40), and a total number of 156 different criteria were identified. No single criterion was common to all hospitals, although nine hospitals employed the same set of criteria as at least one other hospital. The most frequently used criteria are shown in Figure 2.

### Physiological variables

**Figure 3:**
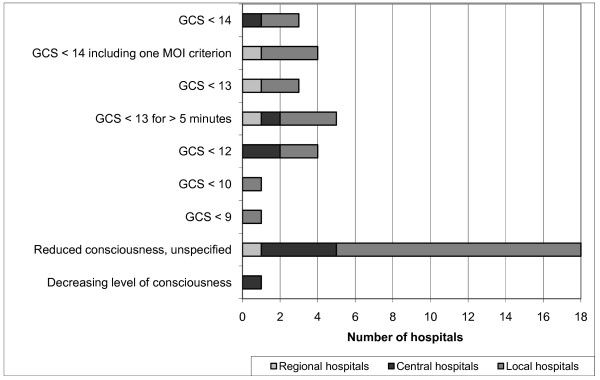
**Distribution of values on the Glasgow Coma Scale (GCS) as a criterion for trauma team activation**.

**Figure 4:**
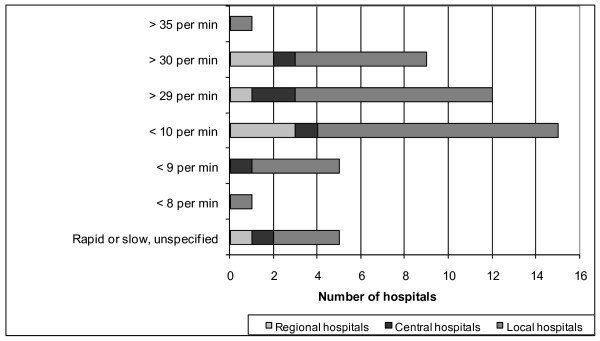
**Distribution of values for ventilation rate (per min) used as a criterion for trauma team activation**.

The two most frequently used physiological criteria were "level of consciousness" ("LEOC") and "hypotension", which were used by 37 hospitals. However, the "cut off" values for LEOC showed considerable variation (Figure 3). Three hospitals used two versions simultaneously: one based on the Glasgow Coma Scale and the other an unspecific criterion called "reduced consciousness". "Hypotension" was either defined as "systolic blood pressure < 90 mmHg", or less specifically as "hypotension", "decreasing blood pressure", or "lack of pulse in the radial artery". Miscellaneous respiratory symptoms was the third largest group, with criteria such as "superficial respiration", "dyspnoea", "stridor", or "airway obstruction". The frequently used criterion, "ventilation rate", also had different cut-off values (Figure 4). "Pulse rate" was used by 20 hospitals, with an upper limit > 120 or > 130 beats per min. Only one hospital specified a lower limit: < 60 beats per min. Other physiologic criteria were "convulsions", "abnormal pupils", "abnormal skin color", "delayed capillary refill", "hypothermia", and "low oxygen saturation". Three hospitals included "Trauma Score" (TS) or "Revised Trauma Score" (RTS) as one of their TTA criteria, with cut off values of < 9 (TS, range 1-16) or < 11 and < 12 (RTS, range 0-12), respectively [21,22].

### Anatomic injury

"Penetrating injury" was the most frequent anatomic criterion, as reported by 43 hospitals. This was often specified as a gunshot wound or stab wound to the torso. "Burn injury" was the second most frequent criterion, but it was unspecified or referred to a variable percentage of the affected body surface: 10, 15, and 20% were all in use. "Inhalation injury" was often included. "Two large fractures", "crush injury", "pelvic injury", and "flail chest" were also frequently used. "Injury to at least two body sections", "impression fracture and "voltage injury" were other criteria used. "Thoracic pain after trauma", "pneumothorax", and "suspected femur fracture" each occurred at only one hospital.

### Mechanism of injury

**Figure 5:**
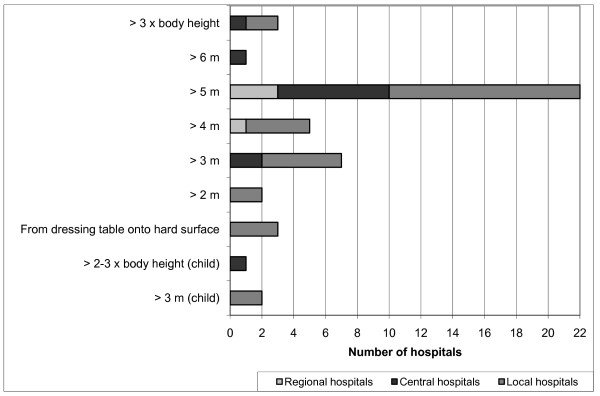
**Distribution of values for fall height (m) when used as a criterion for trauma team activation**.

**Figure 6:**
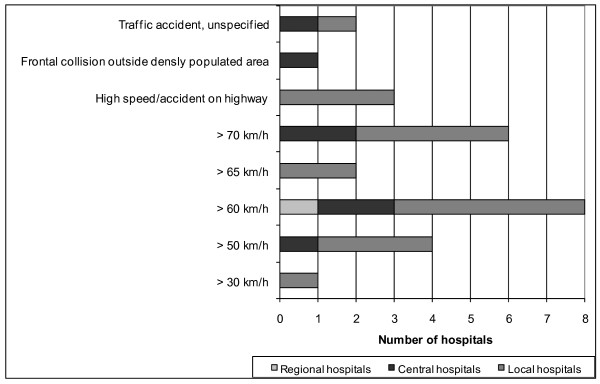
**Distribution of values for crash speed (km/h) when used as a criterion for trauma team activation**.

As an independent criterion, mechanism of injury (MOI) was employed by 38 hospitals (83%) as a reason for activation of the full trauma team. "Fall injury" was the most frequently used criterion and was used in all of these hospitals (but with varying heights; Figure 5). Two hospitals used two heights simultaneously: one used both 4 and 5 m; another both 5 m and/or three times the body length. "Thrown out of vehicle", "death of another individual involved in the accident", "prolonged extrication time", and "pedestrians or cyclists involved in the accident" were other frequent criteria. "Prolonged extrication time" with different specified durations were found in four hospitals. Further, various mechanisms and speeds were used for the criterion "motor vehicle accident" (Figure 6). "Damaged vehicle", "rollover", "crush injury", "explosion", and "avalanche", as well as other unspecific trauma scenes, were used as criteria at several hospitals. "Extreme sport accident" and "industrial accident" each occurred at only one hospital.

### Other criteria

Five hospitals reported simultaneous admission of "more than one trauma patient" as a criterion for TTA. Another hospital operated with "more than two trauma patients" and one with "more than three trauma patients" received at the same time. Seven hospitals also had "transfer of a trauma or unstable patient from lower treatment level" as a TTA criterion. "Drowning" was used as criterion at seven hospitals, while three hospitals would activate their trauma team "when air ambulance physician requests TTA".

### Pediatric cut-off values

"Pediatric trauma" was found as an independent criterion for TTA in two hospitals. Otherwise, specific cut-off values for children were applied to the criteria "burn injury" (> 10% of the body surface), "hit by motor vehicle" (speed > 30 km/h), and "fall injury" (height > 3 m or > 2-3 times the child's body length).

### Relative criteria

In most hospitals, the presence of a single criterion results in TTA, although in some, they consider the use of "relative" or additional criteria for TTA. These are mainly based on MOI, age, pregnancy, and patient co-morbidity. Some hospitals used these as "absolute" criteria; others used them to simply lower the threshold. Three hospitals had criteria based on MOI that were to be considered "in combination with identified symptoms or injuries and clinical aggravation of vital parameters". One hospital used relative criteria implicating that the surgeon on call decided activation or not. Another hospital required at least two relative criteria for TTA. Here, the MOI criteria were not valid if some time had passed, and the patient remained almost unaffected.

### Tiered response

Two hospitals used relative criteria for the activation of a modified (limited) trauma team. Another hospital reported separate criteria for calling the team leader, who was informed about the accident and then decided whether to activate the full or modified trauma team. Thus, at least three hospitals in Norway operated with tiered trauma team activation. One hospital also used separate criteria for calling other medical specialists beyond the ordinary team members.

## Discussion

The main finding in this survey was a conglomerate of criteria for trauma team activation, as well as widely different physiological cut-off values.

A limitation of this study is that the collected information is based on a single eligible contact person. Verification of the answers given in the performed interviews was not attempted, e.g., by interviewing other persons within the same hospitals.

In 2000, 52 hospitals in Norway admitted potentially severely injured patients, and this number was reduced to 49 hospitals in 2008 [18,23]. Isaksen et al. (2006) noted an increase in the implementation of predefined trauma teams (88% in 2004 vs. 52% in 2000) and predefined TTA criteria (29 of 44 hospitals in 2004 vs. 19 of 27 hospitals in 2000) [18,23]. Although the qualitative contents of these developments was not assessed previously, we can now document a further increase of 98% of hospitals having a defined trauma team and 96% having TTA criteria in 2008.

To translate the significance of an injury identified in the field to in-hospital use, many systems use a variation of the ACS-COT field triage scheme as their TTA-decision scheme [4-10]. This scheme and subsequent revisions were initially intended to guide pre-hospital personnel to identify the most severely injured patients. Many criteria in the ACS-COT triage scheme, when used as a single criterion, lack high sensitivity for severe injuries [7]. Indeed, several criteria have been deemed anecdotal or of unproven predictive ability [7].

Physiologic criteria possess significantly higher sensitivity and better positive predictive values (PPVs) in identifying those severely injured [24,25]. PPV is understood as the percentage of severely injured patients among all patients who receive TTA. The classical concept of "specificity", defined as the probability of no TTA among those with minor injury, gives little information about "unnecessary" strain to the trauma team. This is because it takes into account the large number of patients with minor injuries for whom TTA is never considered [20]. Over-triage rates of 25-50% and under-triage rates of 0-5% are seen as acceptable, but it is reasonable to attempt to reduce these rates further [9].

In several studies, MOI criteria have demonstrated poor performance when employed alone to detect severe injury, and the removal of many has been suggested [20,26-28].

In our study, 83% of included hospitals used MOI as an independent criterion for full TTA, despite the substantial amount of evidence suggesting its low accuracy. Some studies indicate that it is useful to limit criteria to only those that are scientifically documented, thereby reducing over-triage without increasing under-triage [29,30]. In a study by Cook et al., the number of criteria for full TTA was reduced to incorporate only physiological and anatomic variables. This resulted in less over-triage without compromised safety [31].

A core issue in the original ACS-COT scheme is a set up of weighted steps using physiologic (Step 1 - potential critical injury), anatomic (Step 2 - potential serious injury), MOI criteria (Step 3 - potentially severe but occult injury), and special considerations (Step 4 - underlying conditions and comorbidity) [4-10]. The motivation was to prevent under-triage of patients not showing vital derangement immediately following the accident. Our findings, however, revealed a non-differentiated use according to the nature of the criteria (physiologic, anatomic, and MOI), in which a single criterion was often used to activate the trauma team. A single criterion may cause low accuracy and should preferably be seen in conjunction with other criteria to increase triage accuracy [20]. Additionally, vague or unspecific criteria (e.g., abnormal respiration and decreased consciousness) may be interpreted rather differently by the personnel involved.

Studies from Great Britain, Denmark, and Australia have shown wide variation with respect to TTA criteria within the same country and region, despite comparable trauma populations [32-34]. As evident in our study, it is not clear why hospitals choose different "cut-offs", but tradition rather than evidence was cited as a possible explanation [30,31]. We found that hospitals in Norway mainly use TTA criteria based on a combination of experience from other hospitals, local adjustments, and expert opinions in their own trauma organization. Where the decision to perform TTA occurs and how different hospitals accommodate pre-hospital information was not investigated in our study.

Few hospitals possess trauma registries and are therefore unable to revise criteria according to their own actual experience. Furthermore, most hospitals admit too few trauma patients to develop evidence-based criteria on their own, suggesting the need for a national consensus. In 2006, the majority (78%) of Norwegian hospitals reported less than 150 annual trauma calls [16]. Of course, over-triage may have positive effects (e.g., training for the trauma team) but is a challenge for hospitals with frequent TTA. Substantial over-triage rates are common in Norwegian referral centers [20,35,36]. While over-triage mainly causes negative system management effects, under-triage is of the greatest concern, as this may cause delayed diagnosis and/or treatment of potentially life-threatening injuries.

Tiered trauma response has evolved as many systems have struggled to cope with an increasing rate of over-triage. Using several response levels, multispecialty teams (when severe injuries and abnormal vital signs are identified), and smaller teams for stable trauma patients promote better resource utilization [37]. In patients with minor to moderate injury, rapid trauma workup is still important, as occult injuries may exist, but it may still not mandate a full trauma team.

## Conclusions

In recent years, Norwegian hospitals have gradually established trauma teams and criteria for the activation of these teams. These criteria show considerable variation, including physiological "cut-off" values.

## Competing interests

The authors declare that they have no competing interests.

## Authors' contributions

KTL and OU conceived this study. KTL, OU, and ES designed the study. KTL performed the telephone interviews and collected data. KTL prepared the figures and conducted the data analysis. KTL and OU drafted the manuscript. All authors interpreted the data and critically revised the manuscript. All authors read and approved the final manuscript.
